# The ecdysone receptor regulates several key physiological factors in *Anopheles funestus*

**DOI:** 10.1186/s12936-022-04123-8

**Published:** 2022-03-19

**Authors:** Surina Maharaj, Elodie Ekoka, Erica Erlank, Luisa Nardini, Janette Reader, Lyn-Marie Birkholtz, Lizette L. Koekemoer

**Affiliations:** 1 Wits Research Institute for Malaria, School of Pathology, Faculty of Health Sciences, University of the Witwatersrand, 7 York Road, Parktown, Johannesburg, South Africa; 2 Centre for Emerging, Zoonotic & Parasitic Diseases, National Institute for Communicable Diseases, Johannesburg, South Africa; 3 Department of Biochemistry, Genetics and Microbiology, Institute for Sustainable Malaria Control, University of Pretoria, Pretoria, South Africa

**Keywords:** 20E-signaling, African malaria vector, *Plasmodium falciparum*, RNA interference

## Abstract

**Background:**

Malaria is a devastating disease, transmitted by female *Anopheles* mosquitoes infected with *Plasmodium* parasites. Current insecticide-based strategies exist to control the spread of malaria by targeting vectors. However, the increase in insecticide resistance in vector populations hinder the efficacy of these methods. It is, therefore, essential to develop novel vector control methods that efficiently target transmission reducing factors such as vector density and competence. A possible vector control candidate gene, the ecdysone receptor, regulates longevity, reproduction, immunity and other physiological processes in several insects, including malaria vectors. *Anopheles funestus* is a prominent vector in sub-Saharan Africa, however, the function of the ecdysone receptor in this mosquito has not previously been studied. This study aimed to determine if the ecdysone receptor depletion impacts *An. funestus* longevity, reproduction and susceptibility to *Plasmodium falciparum* infection.

**Methods:**

RNA interference was used to reduce ecdysone receptor expression levels in *An. funestus* females and investigate how the above-mentioned phenotypes are influenced. Additionally, the expression levels of the ecdysone receptor, and reproduction genes lipophorin and vitellogenin receptor as well as the immune gene, leucine rich immune molecule 9 were determined in ecdysone receptor-depleted mosquitoes using quantitative polymerase chain reaction.

**Results:**

Ecdysone receptor-depleted mosquitoes had a shorter lifespan, impaired oogenesis, were less fertile, and had reduced *P. falciparum* infection intensity.

**Conclusions:**

Overall, this study provides the first experimental evidence that supports ecdysone receptor as a potential target in the development of vector control measures targeting *An. funestus*.

**Supplementary Information:**

The online version contains supplementary material available at 10.1186/s12936-022-04123-8.

## Background

Malaria is a severe disease transmitted by female *Anopheles* mosquitoes infected with protozoan *Plasmodium* parasites. In 2019, a concerning 229 million malaria cases and 409,000 deaths were recorded [1]. Malaria is endemic to tropical and sub-tropical regions of the world. Africa is severely affected, with more than 90% of cases and mortalities occurring on the continent annually [1]. The main malaria vectors in sub-Saharan Africa are either members of the *Anopheles gambiae* complex or the *Anopheles funestus* group [2]. The *An. funestus* group comprises eleven African species, but contains a single significant malaria vector, *An. funestus* [3–6]. Due to its highly anthropophilic (preference for a human host) and endophilic (preference to rest indoors) nature, *An. funestus* is one of the main vectors in sub-Saharan Africa [2, 7].

Efforts to eradicate malaria have been implemented by the World Health Organization (WHO) in the form of The Global Technical Strategy for Malaria (GTS) 2016–2030 [8]. The GTS milestones aim to eliminate at least 90% of malaria by 2030 especially in countries that were most affected in the year 2015 [8]. Unfortunately, the 2020 milestones were not achieved due to several factors [9], emphasizing the need for novel and effective control strategies. Malaria is controlled by adopting strategies targeting either the *Plasmodium* parasite or *Anopheles* vector.

*Anopheles* mosquitoes are primarily controlled using two insecticide-based control interventions: indoor residual spraying (IRS) and long-lasting insecticidal nets (LLIN). These methods are widely employed and their popularity is attributed to their efficiency, cost-effectiveness and ease of implementation [10]. In Africa, LLINs and IRS have proved to be very effective forms of vector control, preventing malaria transmission by 68% and 13% respectively between the years 2000–2015 [11]. Unfortunately, vectors have undergone adaptations that have hindered efforts in malaria eradication. Specifically, *An. funestus* mosquitoes have developed genetic insecticide resistance mechanisms [12–17], physical changes that limit insecticide uptake [18] and behavioural adaptations, such as diurnal and outdoor feeding [19, 20], to evade the current insecticide based interventions. Intensified efforts in vector control are, therefore, required to achieve the WHO targets and potentially eradicate malaria in endemic areas. One solution could be to explore the development of alternative control methods.

There is a growing interest in genetically-based control methods due to the availability of *Anopheles* genomes [21, 22] and development of molecular techniques, such as gene editing by clustered regularly interspaced short palindromic repeats (CRISPR) Cas9 technology [23] among others. The identification of potential genes involved in vector susceptibility to *Plasmodium* or other factors, such as vector longevity and reproduction would be ideal for genetically-based control interventions to reduce malaria transmission. One such gene is the ecdysone receptor (EcR). The EcR is a nuclear receptor that functions as a ligand controlled transcription factor [24]. The EcR binds to the Ultraspiracle protein (USP) and the 20-hydroxyecdysone (20E) hormone to form the EcR-USP complex [25–27]. Together the functional EcR-USP complex binds to a region of DNA either in the form of an inverted or direct repeat, termed the ecdysone response element (EcRE) to activate transcription of the target gene identified as an ‘’early gene’’ [24, 28, 29]. Upon activation, these early genes subsequently regulate the expression of several late genes. This process is termed the 20E signalling pathway. Importantly, EcR via the 20E pathway regulates several physiological functions that ultimately affect malaria transmission in vector species [30].

Vector longevity, reproduction and susceptibility to *Plasmodium* are all factors that affect the density of vectors and/or their competence to transmit malaria [31]. The 20E signaling pathway influences the survival of vectors and treatment of vectors with 20E agonists reduced their longevity [32, 33]. The role that EcR depletion plays in the 20E pathway, i.e. impairing the pathway, by regulating vector longevity however has not yet been characterized in *An. funestus*. Manipulation of this hormone receptor has provided invaluable insights about the 20E signaling pathway in the mosquito. Egg development is hindered and a decrease in the number of eggs developed is observed in *EcR* depleted *Aedes aegypti* and *An. gambiae* mosquitoes due to decreased 20E signaling [34, 35]. Two genes, Lipophorin (*Lp*) and Vitellogenin receptor (*VgR*), regulated by the 20E pathway, largely regulate the reproductive processes vitellogenesis and oogenesis [35–39] therefore to determine if EcR regulates these genes through 20E signaling is of interest. Furthermore, *EcR* depleted *An. gambiae* also developed fewer *P. falciparum* oocysts in comparison to the controls [35]. Implying that functional EcR is required in the 20E signaling pathway, to induce a successful immune response in vectors against *Plasmodium*. An important gene associated with *Plasmodium* immunity, leucine rich immune molecule 9 (*LRIM 9*), regulated by EcR in *An. gambiae* was discovered to exhibit immunity against *Plasmodium berghei* in a previous study [40]. The current study therefore aimed to (i) characterize the role of EcR in longevity, (ii) explore its function in fertility, oogenesis and regulation of *Lp* and *VgR* genes and (iii) to determine its role in LRIM 9 regulation and immunity against *P. falciparum,* all in an important African malaria vector, *An. funestus*.

## Methods

### Biological material

A laboratory strain of *An. funestus* mosquitoes originating from Mozambique (FUMOZ) was used in this study. Female FUMOZ mosquitoes were used for all data collection purposes and were aged accordingly. The FUMOZ strain was reared in the Maureen Coetzee insectary at the Wits Research Institute for Malaria (WRIM) under standard insectary conditions at ± 26 °C with ± 80% humidity and a 12:12 day:night cycle [41].

Gametocytes were produced as per Reader et al. [42]. Briefly, *Pf*NF54 gametocytes were induced from a highly synchronized (> 95%) asexual ring-stage population at 0.5% parasitaemia and 6% haematocrit. Medium was aspirated daily and replaced with fresh medium pre-warmed to 37 °C. Gametocyte health was monitored with microscopy of Giemsa-stained smears. Mature *Pf*NF54 gametocytes (> 98% stage V, 1.5−2.5% gametocytaemia) were used to infect *An. funestus*.

### RNA extraction, DNase I treatment, and cDNA synthesis

RNA was extracted using the TRIzol™ Plus RNA Purification Kit (12183555, Invitrogen, USA) according to the manufacturer's instructions. RNA quality control consisted of spectrophotometry using the Nanodrop One (ND-ONE-W, Thermo Scientific, MA, USA) to determine RNA concentration and purity as well as agarose gel electrophoresis to confirm RNA integrity. A DNase treatment with the TURBO DNA-*free*™ Kit (AM1907, Ambion, TX, USA) was conducted according to manufacturer’s instructions to remove any contaminating genomic DNA carried over from RNA extraction. Once RNA was free of genomic DNA, First strand cDNA synthesis was conducted with the iScript™ cDNA Synthesis Kit (1708891, BioRad, CA, USA).

### Quantitative PCR

Each 1X qPCR reaction consisted of 5 µl of IQ™ SYBR super-mix (1708880, Bio-Rad, CA, USA), 300 nM of each primer, 1 µl of cDNA and nuclease free water to make up a 10 µl reaction. Cycling conditions consisted of an initial denaturation at 94 °C for 3 min, 35 cycles of denaturation, annealing and extension at 94 °C for 20 s, annealing temperature determined by optimization for 25 s and 72 °C for 30 s respectively with a final extension step at 72 °C for 10 min. A melt peak analysis and a no-template control excluding cDNA were included to assess for specificity and contamination, respectively. Reference genes *RPS7* and *RPL19* were used to normalize samples. Primer sequences are listed in Table 1.

**Table 1:** Primer information

Name	Sequence (5’-3’)	Tm (°C)	Amplicon size (base pairs)	Reference
*An. funestus EcR* (forward)	GAT TCT TCC GAC GTA GTG TG	60	484	Designed in this study
*An. funestus EcR* (reverse)	TCC TCG TTG GGT GAG TTA	60		
*An. funestus* T7-*EcR* (forward)	TAA TAC GAC TCA CTA TAG GGA GAG ATT CTT CCG ACG TAG TGT G	67	530	Designed in this study
*An. funestus* T7-*EcR* (reverse)	TAA TAC GAC TCA CTA TAG GGA GAT CCT CGT TGG GTG AGT TA	68		
T7-*GFP* (forward)	TAA TAC GAC TCA CTA TAG GGA GAA CGT AAA CGG CCA CAA GT	66.5	544	Designed by Ms. E. Ekoka (WRIM)
T7-*GFP* (reverse)	TAA TAC GAC TCA CTA TAG GGA GAG GGT GTT CTG GTA GTG	68.4		
qPCR *EcR* (forward)	GCC GGT AGC ACA AGT AAT AG	60	130	Designed in this study
qPCR *EcR* (reverse)	GAT CGA GCA TTC CGA CAG	60		
*LRIM9* (forward)	CAG TTC TTC ACC GCA TAG TT	60	117	Designed in this study
*LRIM9* (reverse)	TTG TCG TCC AGG TAG AGT T	60		
*Lp* (forward)	GCT TCG ACA AGG TGT TAG AG	60	104	Designed in this study
*Lp* (reverse)	AAG ACC AAG AGC GGT AGT	60		
*VgR* (forward)	TAC TTA CGG CGG GAC TTA T	60	147	Designed in this study
*VgR* (reverse)	GGA GCT GAT CCT GTA TGA TTG	60		
*RPS7* (forward)	TTA CTG CTG TGT ACG ATG CC	60.4	134	Amenya et al*.* [14]
*RPS7* (reverse)	GAT GGT GGT CTG CTG GTT	62.3		
*RPL19* (forward)	GAA ACA CCA ACT CCC GAC A	60.2	223	Spillings et al*.* [6]
*RPL19* (reverse)	TCA ACA GGC GAC GCA ACA CA	62.3		

### RNA interference

The starting material for the production of dsRNA molecules consisted of a plasmid containing *GFP* or for *EcR*, cDNA prepared from RNA using the iScript™ cDNA synthesis kit and subject to PCR to obtain a 484 bp amplicon. Total RNA for this purpose was extracted from female *An. funestus* at 24 h post-blood meal, since this is the peak of *EcR* expression [43]. Subsequently, to add T7 promoter sequences to the 5′ end of the *GFP* clone and *EcR* template, a PCR was conducted using the T7-containing RNAi primers and the *GFP* or *EcR* template. The T7-containing 544 bp GFP and 530 bp EcR templates were concentrated and purified with ethanol precipitation to a final concentration of 1 µg/µl. Double stranded RNA molecules homologous to the *EcR* and *GFP* templates were synthesised with the MEGAscript® RNAi Kit (AM1626, Thermo Scientific, MA, USA) according to the manufacturer’s instructions. Specific ds*RNA* products were then concentrated by precipitation to a final concentration of 10 µg/µl. All primers used are described in Table 1.

### dsRNA delivery

To deliver ds*RNA* molecules to mosquitoes, nanoinjection was conducted using the Nanoject II (3-000-204, Drummond, AL, USA). All mosquitoes were fed on a 10% (w/v) sucrose solution prior to nanoinjection. Mosquitoes were cold anaesthetized prior to nanoinjection in the thorax with approximately 500 ng dsRNA. After nanoinjection, mosquitoes were placed in recovery cages where they were provided with a 10% (w/v) sucrose solution and kept under standard insectary conditions to be used for further assays. An uninjected control was included in the biological assays to determine if the phenotypes observed occurred as a result of the nanoinjection procedure.

### Longevity assay

One day old female *An. funestus* mosquitoes were administered with either ds*EcR* or ds*GFP* (control) according to the nanoinjection method described previously. Post injection mosquitoes were provided with a 10% sucrose solution and maintained under standard insectary conditions. Mortality among mosquitoes was recorded each day until all the mosquitoes had died. Three biological replicates were conducted. Knockdown of *EcR* was confirmed with qPCR. Approximately fifteen mosquitoes from each biological replicate were randomly selected from each treatment group 48 h post injection and subject to RNA extraction, RNA quality control, DNase treatment, cDNA synthesis and qPCR as described previously. Knockdown of *EcR* was evaluated at the 24, 48 and 72 h time points to determine the most suitable time to confirm ds*EcR* treatment was successful (Additional file 1: Fig. S1).

### Fecundity assay

Forty newly emerged males and forty newly emerged females were combined into 17.5 cm^3^ BugDorm cages (BD41515, MegaView Science, Taiwan, China) per replicate and allowed to mate for 12 days to achieve the optimal mating success rate (Additional file 2: Fig. S2). Six biological replicates were conducted. Mosquitoes were maintained on a 10% sucrose solution under standard insectary conditions for the duration of this experiment. When females were 10 days old they were isolated prior to ds*RNA* treatment. After injection, the females were placed back with males for the remaining two days to fulfil the 12 day mating period. On the eleventh day, the 10% sucrose solution was removed and replaced with distilled water to encourage blood feeding. On the twelfth day, fifteen females from each treatment group were removed for RNA extraction and qPCR to confirm knockdown. Simultaneously, females were blood fed for 1 h on bovine blood with the Hemotek artificial membrane feeding system. Blood fed females were subsequently removed and placed into individual 250 ml oviposition cups containing approximately 10 ml of distilled water and lined with filter paper over a black background. The number of eggs laid by mated females were counted by microscopy to determine fecundity. Females that had oviposited were dissected after oviposition and their spermathecae observed by microscopy at 200× magnification to determine mating status; virgin females were subsequently excluded from the study. If mosquitoes had not oviposited seven days post blood meal, they were dissected to determine their mating status and observe if eggs were retained in ovaries.

### Plasmodium infection assay

*Anopheles funestus* females aged seven to ten days old (to allow for optimal mating and encourage blood feeding) were injected with 10 µg/µl of ds*EcR* or ds*GFP* as described previously. Ninety mosquitoes were included per replicate and a total of eight biological replicates were conducted. Subsequent to injection, the 10% sucrose solution was removed and replaced with distilled water to encourage blood feeding. Concurrently, mosquitoes were isolated for RNA extraction, DNase 1 treatment, cDNA synthesis and qPCR. Twenty-four hours after nanoinjection, mosquitoes were offered a *Pf*NF54 infected blood meal (> 98% stage V gametocytes, 1.5−2.5% gametocytaemia, 50% (v/v) A + male human serum, Interstate blood bank Inc, Memphis, Tennessee, USA) for 40 min using a glass feeder. Unfed mosquitoes were discarded while fed mosquitoes were maintained on a 10% sucrose solution for eight days post feeding. After this time, mosquitoes were aspirated into ethanol to immobilize them and subsequently transferred to 1× PBS. Mosquito midguts were dissected 8 days post infection and stained on a microscope slide using 0.1% mercurochrome (M7011, Sigma, MO, USA) [44]. Midguts were subsequently viewed under a compound microscope between 20 and 40× magnification and the intensity and prevalence of oocysts in each midgut was counted and recorded.

### Statistical analysis

All qPCR data were analysed using relative gene expression analysis as per 2^−∆∆Ct^ method [45]. The 2^−∆∆Ct^ values were subsequently log transformed and statistical significance was determined using an unpaired student’s t-test [46]. The Kaplan Meier survival curve was used for longevity analysed using the Log-Rank test to calculate statistical significance (GraphPad Prism 8). Statistical analysis for the eggs oviposited, fertility and *P. falciparum* oocyst intensity were evaluated for normality using the Shapiro–Wilk test. If data were not normally distributed the Mann–Whitney test was used, otherwise an unpaired students t-test was used. To determine statistical significance for the number of ovaries containing mature eggs compared to those with immature follicles, a two tailed Fisher’s exact test was used. *Plasmodium falciparum* oocyst prevalence data was analysed using a Chi-squared test. For all analyses, *p* values < 0.05 were considered to be statistically significant.

## Results

### Injection of ds*EcR* reduces *EcR* expression but does not influence *Lp*, *VgR* and *LRIM 9* expression in *An. funestus*

**Fig. 1:**
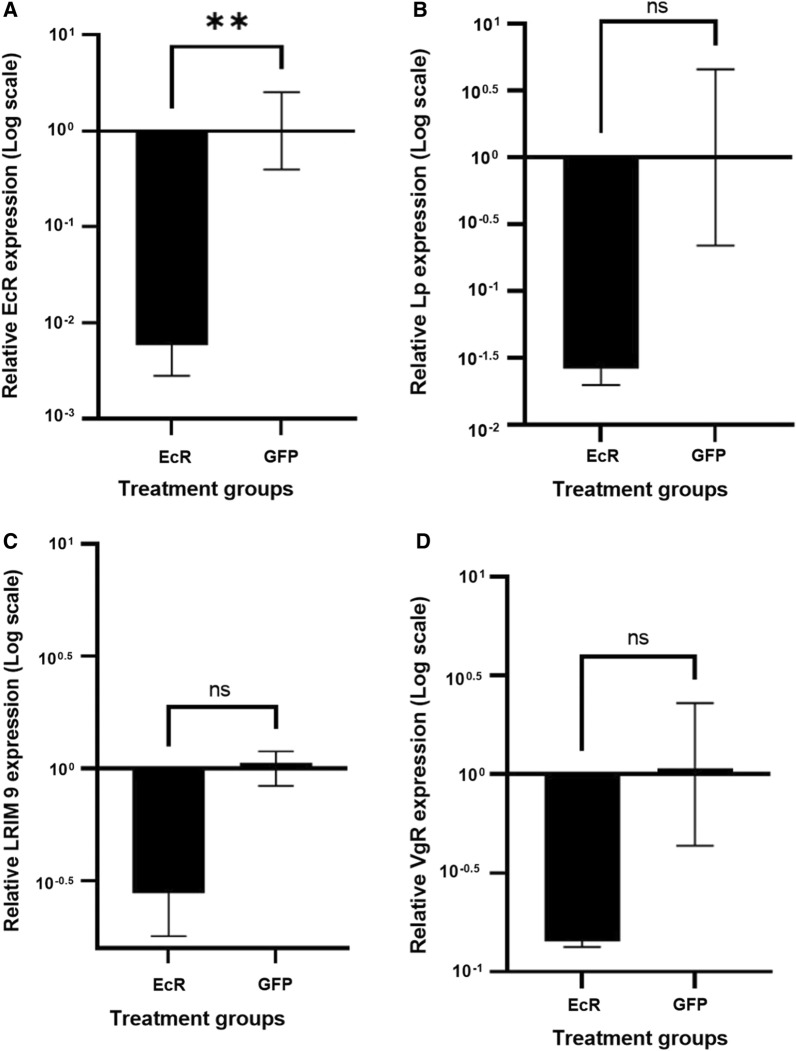
Log transformed relative fold change normalized against reference genes for *EcR*, *Lp*, *LRIM 9* and *VgR* genes in ds*EcR* treated female *An. funestus* compared to the ds*GFP* treated control. Depletion of *EcR* resulted in the significant downregulation of *EcR*. The Lp, *LRIM 9* and *VgR* genes were not significantly downregulated upon depletion of EcR. Error bars indicate standard error of mean (SEM). ** denotes *p* < 0.01 and ns = not statistically significant. At least three biological replicates were used for each gene.

To assess different phenotypes associated with *EcR* depletion, *An. funestus* females were injected with dsRNA targeting *EcR* (ds*EcR*) alongside ds*GFP* as a control. Relative expression, as determined by qPCR, was used to confirm gene silencing. *EcR* was significantly downregulated (*t*(6) = 4.3, *p* = *0.0051*, unpaired student’s t-test) with a mean expression ± SEM of − 2.23 ± 0.32 fold in dsEcR injected samples compared to − 1.50e−006 ± 0.40 fold confirming that the ds*EcR* injection significantly reduced *EcR* transcription (Fig. 1A).

Likewise, the expression of several other transcripts *Lp*, *VgR and LRIM 9* previously demonstrated to be involved in mosquito reproduction, or susceptibility to *Plasmodium* species [38, 40, 47], were measured. In ds*EcR* injected females, *Lp* expression was downregulated to a mean ± SEM of − 1.58 ± 0.12 fold compared to 1.33e−006 ± 0.66 fold in ds*GFP* injected females (Fig. 1B). This difference was however not statistically significant (t(4) = 2.35, *p* = 0.0785, unpaired student’s t-test). Expression of *LRIM 9* was reduced to a mean ± SEM of − 0.56 ± 0.19 fold in dsEcR injected females compared to dsGFP injected females with a mean ± SEM of − 1.00e−006 ± 0.08 fold, however no statistical significance was observed (t(4) = 2.703, *p* = 0.0539, unpaired student’s t-test) (Fig. 1C). Moreover, *VgR* expression was depleted to a mean ± SEM of − 0.84 ± 0.03 fold in ds*EcR* treated females compared to − 6.67e−007 ± 0.36 fold in ds*GFP* treated females (Fig. 1D). This difference was not statistically significant (t(4) = 2.327, *p* = 0.0805, unpaired student’s t-test). No significant difference was observed in *Lp*, *LRIM 9* nor *VgR*, suggesting depletion of *EcR* does not affect expression of these genes in this study.

### *EcR* depletion results in decreased longevity in *An. funestus*

**Fig. 2:**
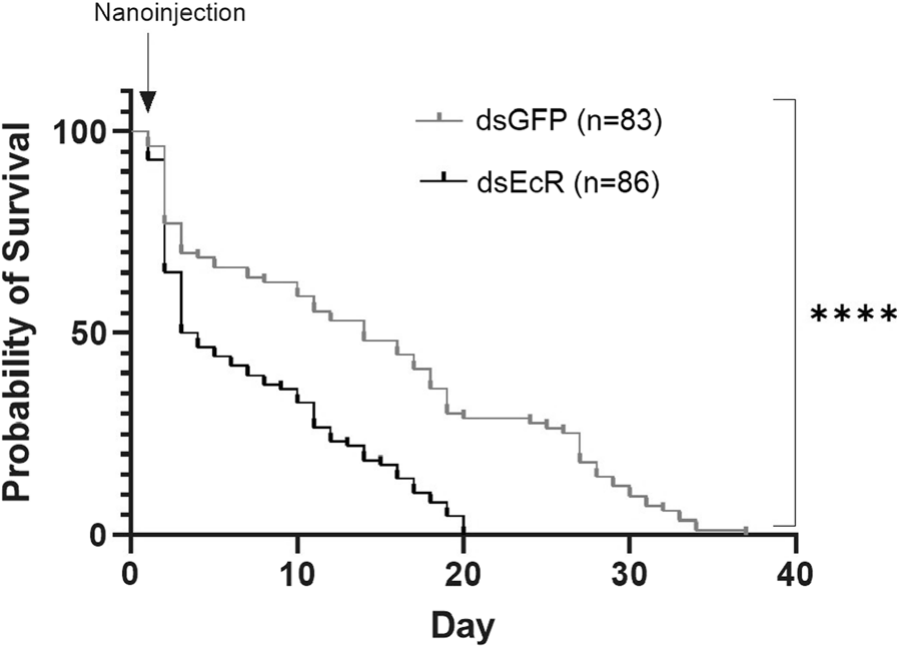
Kaplan Meier survival curve indicates significantly decreased survival in ds*EcR* injected *An. funestus* females compared to ds*GFP An. funestus* females. Statistical significance calculated with the Log rank test, *χ*^2^ = 30.18, DF = 1, *p*˂0.0001. **** denotes *p* < 0.0001. n represents sample size

To determine the effect of *EcR* depletion on *An. funestus* longevity, mortality was recorded daily in ds*EcR* and ds*GFP* treated females until all mosquitoes had succumbed. Mosquitoes injected with ds*EcR* did not survive past 20 days whereas those injected with ds*GFP* survived up to 37 days. There was a statistically significant difference in the probability of survival between the two groups (*χ*^2^_(1, *N*= 240)_ = 30.18, *p * < 0.0001, Log rank test). The median survival (time taken to reach a survival of 50%) of ds*GFP* females was 14 (95% CI of ratio: 2.96–5.41) days compared to 3.5 (95% CI of ratio: 0.19 to 0.34) days for females injected with ds*EcR.* This translates to a 54% decrease in longevity in ds*EcR* injected mosquitoes (Fig. 2). No significant difference (*χ*^2^_(1, *N*= 240)_ = 2.011, *p* = 0.1561¸ Log rank test) was observed between the ds*GFP* and uninjected control groups, suggesting that the nanoinjection procedure did not influence longevity (Additional file 4: Table S1).

### *EcR* depletion decreases reproductive success in *An. funestus*.

**Fig. 3:**
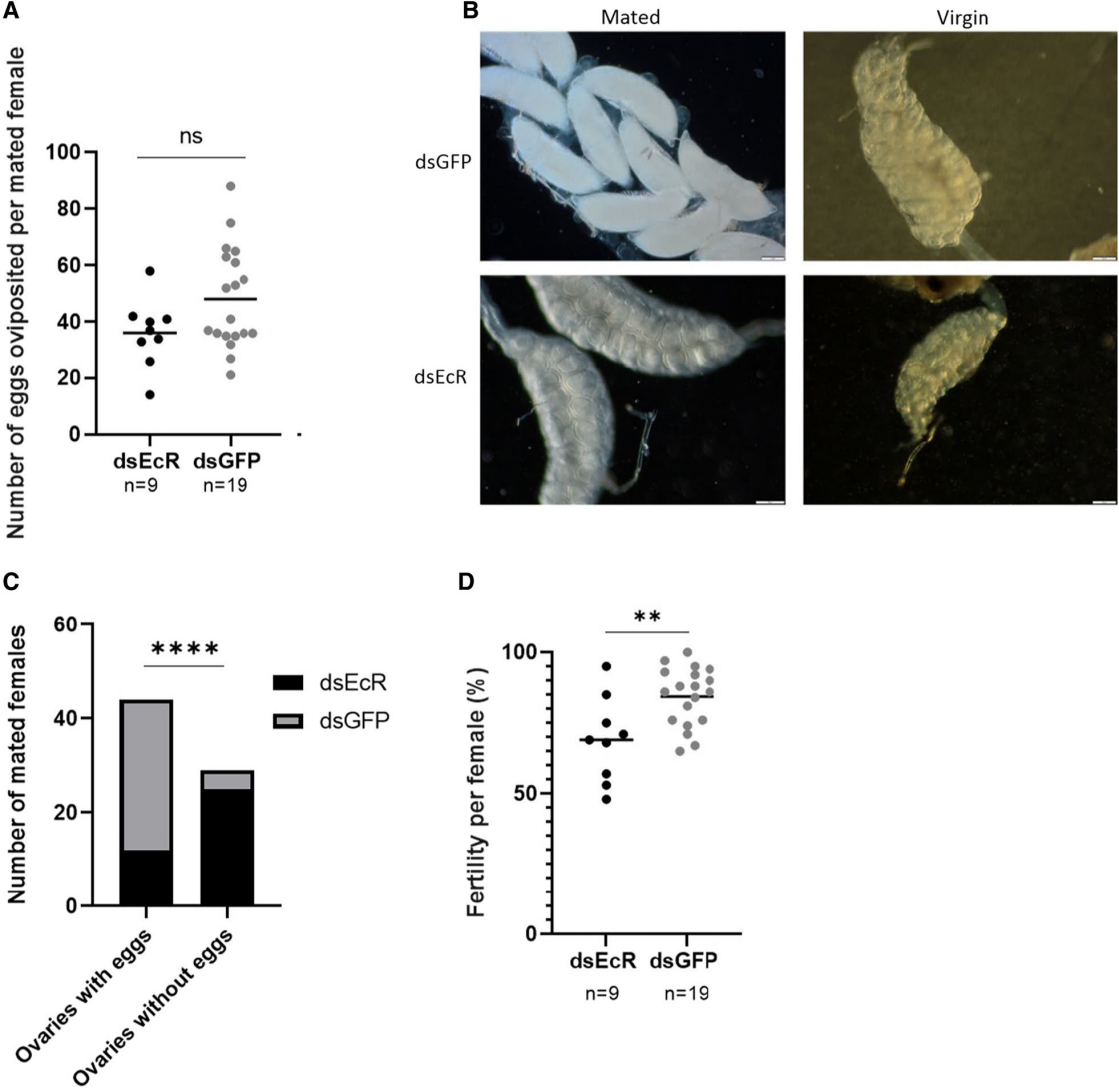
Several reproductive factors are affected when *EcR* is depleted. The median number of eggs oviposited did not differ between ds*EcR* and ds*GFP* (*p* > 0.05). **A** The total number of eggs developed in mated females was significantly decreased in ds*EcR* injected *An. funestus* females compared to ds*GFP An. funestus* females (*p *< 0.0001). **B** The development of eggs differed between mated ds*GFP* controls and mated ds*EcR* treated *An. funestus* females. **C** Mature, developed eggs (ovoid shape) were observed in mated uninjected and ds*GFP* treated *An. funestus* females. Contrastingly, ds*EcR* treated *An. funestus* females contained immature and undeveloped oocytes (spheroid shaped) in their ovaries that more closely resembled the ovaries of virgin *An. funestus* females of all treatment groups. The median fertility of ds*EcR* treated *An. funestus* females was significantly lower compared to ds*GFP An. funestus* females (*p * <  0.01). **D** Error bars represent SEM. ** denotes *p* < 0.05; **** denotes *p* < 0.0001; ns = not statistically significant (*p* > 0.05). n represents sample size

To determine EcRs effect on fecundity, the number of eggs oviposited by mated females was counted in ds*EcR* and ds*GFP* treated females. Although a total number of 157 females were induced to lay eggs, the number of females that oviposited were low. Altogether, 9 dsEcR treated females and 19 dsGFP treated females oviposited. No significant difference was observed in the blood feeding rates between ds*EcR* and ds*GFP* groups (Additional file 3: Fig. S3). The mean number of eggs per female ± SEM oviposited in ds*EcR* treated females was 36.11 ± 4.01 compared to 48.11 ± 4.12 in ds*GFP* treated females (Fig. 3A). This decrease however, was not significant (*t*(26) = 1.813, *p* = 0.0814, unpaired student’s t-test), suggesting that *EcR* depletion does not affect the number of eggs oviposited. Additionally no statistical difference (*t*(91) = 0.1522, *p* = 0.8793, unpaired student’s t-test) was observed between the ds*GFP* and uninjected control groups, suggesting that the nanoinjection procedure had no effect on the number of eggs oviposited (Additional file 4: Table S1). Those mosquitoes that had not oviposited were dissected to observe if eggs were retained in the ovaries. Surprisingly, the majority of ovaries of ds*EcR* injected mated *An. funestus* females closely resembled that of virgin *An. funestus* females, containing immature and undifferentiated oocytes whereas the majority of ds*GFP* injected females ovaries contained mature eggs (Fig. 3B). In ds*EcR* injected females, 32% of mated females developed eggs whereas 89% of ds*GFP* injected females developed eggs. This difference observed was statistically significantly different (*p * < 0.0001, Fisher’s exact test) (Fig. 3C). This suggests that ds*EcR* injected *An. funestus* females typically develop fewer eggs compared to controls. Together these results substantiate that the depletion of *EcR* had a deleterious effect on oogenesis by preventing the development of oocytes into mature eggs in *An. funestus.* No significant difference (*p* > 0.9999, Fisher’s exact test) was observed between the ds*GFP* and uninjected control groups, indicating the nanoinjection procedure did not affect the oviposition process (Additional file 4: Table S1). Although very few ds*EcR* injected females oviposited eggs (n = 9), fertility was also compared between the ds*EcR* and ds*GFP* treated mosquitoes by monitoring the hatching of the eggs oviposited. The median percentage fertility (25–75% percentile) in ds*EcR* treated mated females was significantly lower at 69% (55–80%) compared to 86% (76–93%) in ds*GFP* treated mated females (*t*(26) = 3.169, *p* = 0.0039, unpaired student’s t-test) (Fig. 3D). These results indicate that *EcR* depletion results in *An. funestus* females that are less fertile than control females. Furthermore, no significant differences (*t*(46) = 0.5975, *p* = 0.5531, unpaired student’s t-test) existed between the ds*GFP* and uninjected control groups, corroborating that the nanoinjection procedure did not affect fertility in *An. funestus* (Additional file 4: Table S1). Taken together, results showed that EcR influences reproductive processes such as oogenesis and fertility in *An. funestus*.

### EcR depletion decreases P. falciparum infection intensity in An. funestus.

**Fig. 4:**
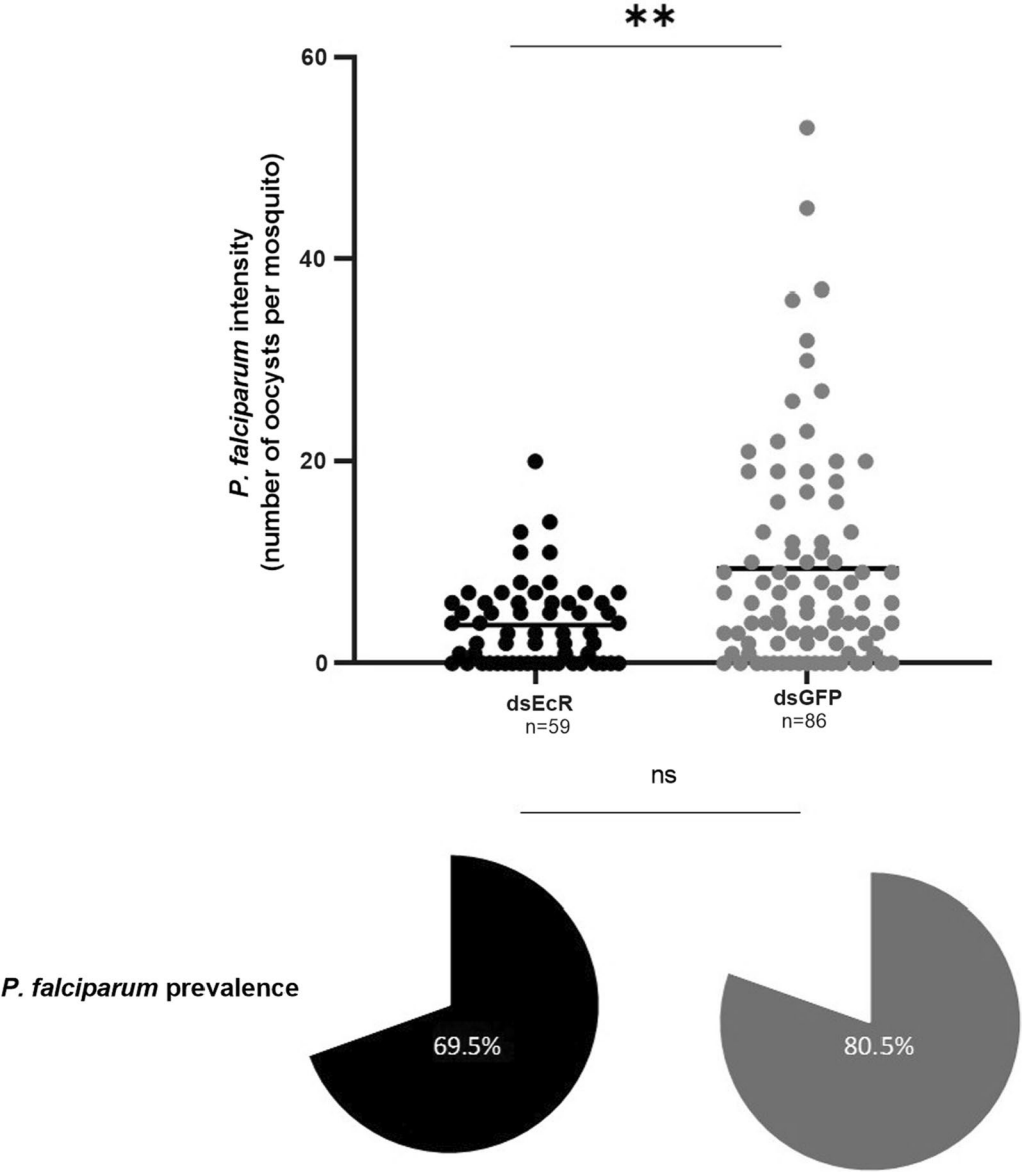
Depletion of *EcR* results in decreased oocyst intensity but not oocyst prevalence in *An. funestus* females. The median number of oocysts in ds*EcR* treated *An. funestus* females was significantly decreased compared to ds*GFP An. funestus* females (*p*˂0.01). The prevalence of infection did not differ statistically between dsEcR and ds*GFP *treated groups (*p* > 0.05). Error bars represent SEM. ** denotes *p* < 0.01; ns = not statistically significant (*p* > 0.05). n represents sample size

The effect of *EcR* depletion on *An. funestus* infection by *P. falciparum* was determined by comparing the intensity of oocysts and the prevalence of infection between ds*EcR*-injected and ds*GFP*-injected mosquitoes. Oocyst intensity was significantly decreased by 50% in ds*EcR* treated *An. funestus* females with 3.80 ± 0.54 oocysts per midgut compared to ds*GFP* treated *An. funestus* females with 9.37 ± 1.17 oocysts per midgut (Mann–Whitney U = 1751, *p* = 0.0013, 8 biological replicates) (Fig. 4). *EcR* depletion significantly decreased the number of oocysts that developed in the midgut. Additionally, the mean prevalence of infected *An. funestus* females was 69.45% (± 6.05) in ds*EcR* injected *An. funestus* females compared to 80.46% (± 4.28) in ds*GFP* injected *An. funestus* females (Fig. 4). No significance in infection prevalence was observed between ds*EcR* and ds*GFP* treated females (*χ*^2^_(1, *N*= 720)_ = 2.205, *p* = 0.1376, Chi-squared test), suggesting that *EcR* depletion does not influence the prevalence of infection by *P. falciparum*. These results corroborate that *EcR* influences *P. falciparum* oocyst development in *An. funestus* but does not affect the incidence of infection by *P. falciparum*. No significant difference was observed between the ds*GFP* and uninjected control groups for either the *P. falciparum* infection intensity (Mann–Whitney U = 2300, *p* = 0.5337) or prevalence (*χ*^2^_(1, *N*= 720)_ = 0.2584, *p* = 0.6112, Chi-squared test), suggesting that the nanoinjection procedure did not influence these processes (Additional file 4: Table S1).

## Discussion

This study provides the first experimental evidence that *EcR* plays a crucial biological role in the major vector species *An. funestus*. Vectors such as *An. funestus* among others require novel control strategies due to the increase in insecticide resistance which reduces the efficacy of current vector control interventions. The transcription factor *EcR* was identified as a potential gene to be investigated due to its pleiotropic effects discovered in mosquitoes [30]. The current study sought out to determine how 20E signaling, using *EcR* as a proxy, regulates several factors that influence vector density and competence in *An. funestus*.

Depletion of *EcR* in *An. funestus* reduced longevity by over a half compared to the control. Similarly, in other insect species such as *Drosophila melanogaster*, *Nilaparvata lugens* and *Sitobion avenae*, reduction of *EcR* using ds*EcR* resulted in decreased longevity [48–50]. Together these findings confirm that reducing transcription of *EcR* results in deleterious effects on longevity, corroborating that EcR is essential in the regulation of longevity. Longevity plays a major role in malaria transmission. Wild *An. funestus* can survive approximately 30 days [2] and in this time are able to complete the extrinsic incubation period (EIP) required for *Plasmodium* parasites to mature and become infectious to humans. Several factors such as temperature, vector species, parasite species, vector nutrition and survival can influence the EIP [51]. Defining the duration of the EIP is therefore difficult, however it typically ranges from 12 to 16 days [51–53]. Reducing *EcR* in *An. funestus* females could therefore decrease longevity to a point where the EIP cannot be completed in the field, thereby reducing malaria transmission. Additionally, decreased vector longevity can reduce the vector to human ratio, possibly reducing malaria transmission rates [31].

Malaria transmission and vector density are also impacted by vector reproduction. Moreover, ingestion of a blood meal (a prerequisite for anautogenous mosquito reproduction) increases the risk of *Plasmodium* transmission. Two reproductive processes, fecundity and fertility were assessed after *EcR* depletion. Impaired oogenesis was observed after reduction of *EcR* levels in *An. funestus*. Consistent with these findings, other studies reported reduced follicle length [34] and a reduction in the number of eggs developed [35] after *EcR* depletion in *Ae. aegypti* and *An. gambiae*. Lp and VgR are two important proteins involved in lipid transport to oocytes and the uptake of the YPP vitellogenin providing nutrients for oocytes to develop respectively [35–39]. In the current study, depletion of *EcR* did not affect the expression of the *Lp* and *VgR* genes, however 20E signaling was confirmed to regulate *Lp* and *Vg* gene expression in other studies [35, 38, 54, 55]. However, these studies are not directly comparable due to differences in methodologies used and in addition discrepancy may be due to the complex interaction of several other genes in the 20E pathway suggesting that EcR is not solely responsible for the regulation of these genes.

The profound mechanism of gene regulation by EcR is not yet fully understood but studies have discovered several key elements of this process. Ecdysone responsive genes such as *Vg* i.e. those containing *EcR* binding sites, also contain binding sites for several other transcription factors which regulate gene expression differentially depending on temporal and spatial requirements [55]. Changes in gene regulation in these ecdysone responsive genes are brought about indirectly by EcR coupled with the action of other transcription factors [56]. However, EcR can also directly regulate the specific temporal or spatial expression of genes [57]. Moreover, ecdysone responsive genes such as *Lp* are regulated differentially in mosquito species such as *Ae. aegypti* and *An. gambiae* through a currently unknown mechanism [35, 38]*.* The current study has limited information on gene regulation by EcR but the above studies confirm that future studies should concentrate on this process to provide a clearer understanding of this process in *An. funestus*.

Depletion of *EcR* significantly reduced fertility in *An. funestus*. A source of uncertainty arises from the small sample size in this study, however it is known that *An. funestus* is refractory to colonization due to low blood feeding rates, mating success and oviposition rates (Koekemoer, pers comm). This experiment should be repeated to provide more clarity. Nevertheless, results from the current study are noteworthy, a haem peroxidase (HPX15) was found to control fertility in *An. gambiae* [58]. This enzyme requires 20E for normal functioning [58] and providing a mechanistic basis for the role of *EcR* in fertility. Silencing HPX15 was found to decrease fertility in females over multiple gonotrophic cycles [58]. EcRs regulation of fertility could, therefore, be due to its function as a transcription factor of HPX15, but will have to be investigated in future.

Furthermore, EcR was found to control transcription of the immune molecule LRIM 9 in a study by Upton et al. [40]. Depletion of *LRIM 9* with ds*LRIM 9* in *An. gambiae* resulted in a threefold higher *P. berghei* oocyst intensity, validating the importance of LRIM 9 in providing immunity against *P. berghei* [40]. However, this was not evident when *EcR* was depleted in *An. funestus* as a decrease in *P. falciparum* oocyst intensity was observed instead. This discrepancy could presumably be due to a variance in the activity of *LRIM 9* against different *Plasmodium* species studied. Similarly, it could be attributed to differences in the immune systems and regulation of *Plasmodium* infection between *An. gambiae* and *An. funestus*. Immune genes of *An. funestus* are genetically more similar to the Asian malaria vector *Anopheles stephensi* than *An. gambiae* due to evolutionary divergences in these vectors [59–61]. The contradiction in these results suggest a different mechanism is involved in decreasing the intensity of *P. falciparum* oocysts when *EcR* is knocked down in *An. funestus*. Similar to the current study, Werling et al. [35] observed a decrease in oocyst intensity when ds*EcR* treated *An. gambiae* was infected with *P. falciparum*. Strikingly, this decrease in *P. falciparum* oocysts was accompanied by larger, faster maturing sporozoites which was brought about by Lp [35]. Presently, limitations such as a decrease in the longevity of ds*EcR* injected females (in this study) limited the sample size of infected mosquitoes available 16–18 days post infection and hindered the investigation of faster maturing sporozoites in *An. funestus*. These findings should however be further investigated in *An. funestus* once limitations are overcome to determine if the reduction in oocyst intensity results in faster maturing sporozoites and a more rapid EIP for *P. falciparum* or if a different immune strategy is adopted by the different *Anopheles* species. If the latter is proven true, an RNAi based approach targeting *An. funestus* EcR will need to be taken into consideration. It will also be interesting to determine the length of time that that *EcR* remains depleted in *An. funestus* after ds*EcR* treatment as this is a key factor in the development for a potential RNAi based control method.

If it is found to be true that depleting *An. funestus EcR* in any way increases parasite infectivity, genetically based control methods, such as gene drives [62], paratransgenesis [63], transmission blocking vaccines [64] or simply any form of *EcR* gene modification cannot be considered. Additionally, it will be important to confirm these findings on wild *An. funestus* populations from different geographical areas. Fortunately, decreasing *EcR* levels is only one of the possibilities to target ecdysone signalling to reduce malaria transmission. Nonsteroidal 20-hydroxyecdysone agonists share structural similarities with 20E, hence they are able to competitively bind to the EcR-USP complex, amplifying 20E signalling and all of its effects [65]. Five 20E agonists, namely chromafenozide, fufenozide, halofenozide, methoxyfenozide and tebufenozide are currently available as insecticides targeting agricultural pests [66, 67]. These 20E agonists are promising as potential malaria vector control strategies as they exhibit low toxicity to non-target organisms and penetrate the mosquito cuticle [66–68]. The efficacy of some of these compounds has also been demonstrated against several *Anopheles* vectors [32, 33, 69].

## Conclusions

The current study provided some insight on the biological function of *EcR* in *An. funestus* as a regulator of longevity, oogenesis, fertility and susceptibility to *P. falciparum*. Further research is required to determine the mode of action of, and genes involved in, EcRs regulation of pathways governing longevity, reproductive success and importantly *Plasmodium* infectivity in *An. funestus*. For example, the complex interaction of EcR and the 20E signalling pathway can further be elucidated through next generation RNA sequencing. This will allow for the identification of the complete profile of early and late genes regulated by EcR, providing a holistic overview of this pathway. These genes can thereafter be examined to determine their functions in regulating mosquito physiological process that potentially target vector density and competence. Subsequently, success in both laboratory and field based aspects of this research could result in approved EcR based vector control methods that will potentially reduce malaria transmission.

## Supplementary Information

**Additional file 1: Figure S1.** Relative *EcR* expression levels in ds*EcR* injected *An. funestus* females compared to ds*GFP* injected *An. funestus* females. The *EcR* gene was knocked down in ds*EcR* injected *An. funestus* females as *EcR* expression levels were drastically reduced compared to the *GFP* control. Statistically significant knockdown was evident in ds*EcR* injected *An. funestus* females 24, 48 and 72 h after injection as *EcR* expression in ds*EcR* injected *An. funestus* females was 0.11 ± 0.006 (*p* < 0.05), 0.01 ± 0.001 (*p* < 0.01) and 0.2 ± 0.06 (*p* < 0.05) respectively when compared to the *GFP* injected control of 1. This data confirmed *EcR* knockdown in *An. funestus* females injected with ds*EcR*. Data is representative of 2 biological replicates and normalised using an average of *RPS7* and *RPL19* reference genes. Expression levels calculated using relative quantification method (∆∆Ct). At each time point statistical significance was assessed with the unpaired student’s t-test. *p < 0.05, **p < 0.01. Error bars represent standard deviation.

**Additional file 2: Figure S2.** The highest mating success rate was achieved when *An. funestus* males and females are combined for 12 days after which no further increases are observed. The percentage mating success rate increased progressively until it reached its highest value of 62.2% after 12 days of mating. After this point, the mating success rate reached a plateau until day 20. Statistical significance was calculated using one-way ANOVA with Tukey’s post hoc analysis to correct for multiple comparison. Data represents the means of 3 biological replicates. Error bars represent standard deviation of means. ns = not statistically significant *p* > 0.05; ** =* p* < 0.01; *** = *p *< 0.001; **** = *p *< 0.0001. (n) = number of females per time point across 3 biological replicates.

**Additional file 3: Figure S3.** Blood feeding rates did not differ between treatment groups. Insignificant differences amongst treatment groups confirmed that the blood feeding rates did not influence any changes observed in the phenotypes of ds*EcR* treated *An. funestus* females (*p* > 0.05). Statistical significance calculated using an unpaired student’s t-test. Error bars represent standard deviation. ns = not statistically significant *p *> 0.05.

**Additional file 4: Table S1.** Statistical significance between ds*GFP* and uninjected controls from the various biological assays conducted.

## Data Availability

The datasets generated and analysed during the current study are available from the corresponding author on reasonable request.

## Abbreviations

20E: 20-Hydroxyecdysone
dsRNA: Double stranded RNA
EcR: Ecdysone receptor
GFP: Green fluorescent protein
IRS: Indoor residual spraying
LLIN: Long-lasting insecticidal nets
Lp: Lipophorin
LRIM 9: Leucine rich immune molecule 9
VgR: Vitellogenin receptor
WHO: World Health Organization
