# Supplementary figures and images for: The ecdysone receptor regulates several key physiological factors in Anopheles funestus
Source: Malar J. 2022 Mar 19;21:97. doi: 10.1186/s12936-022-04123-8 (PMC8934008; doi:10.1186/s12936-022-04123-8)

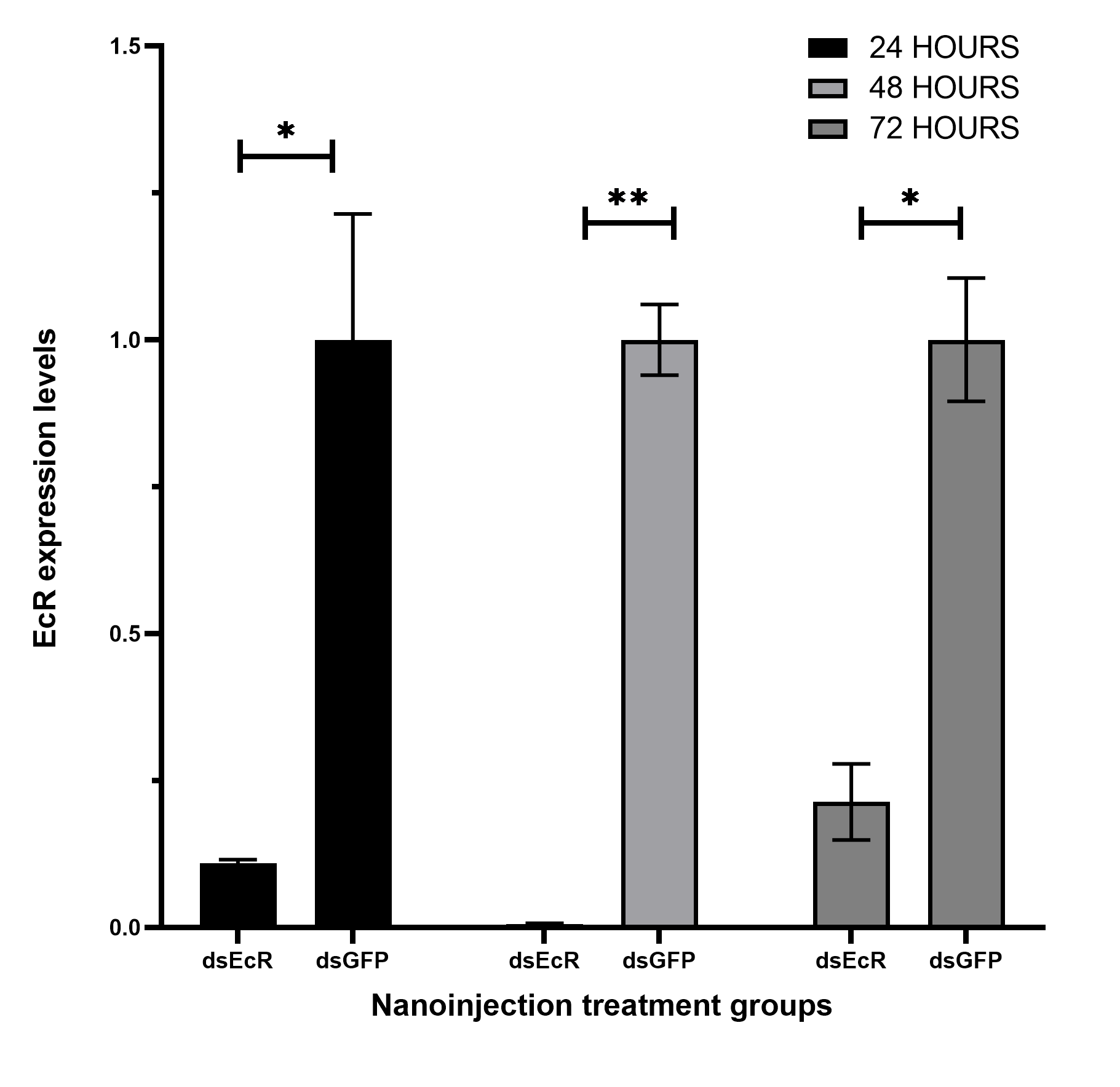

Supplement: Supplementary file 1 — Additional file 1: Figure S1. Relative EcR expression levels in dsEcR injected An. funestus females compared to dsGFP injected An. funestus females. The EcR gene was knocked down in dsEcR injected An. funestus females as EcR expression levels were drastically reduced compared to the GFP control. Statistically significant knockdown was evident in dsEcR injected An. funestus females 24, 48 and 72 h after injection as EcR expression in dsEcR injected An. funestus females was 0.11 ± 0.006 (p < 0.05), 0.01 ± 0.001 (p < 0.01) and 0.2 ± 0.06 (p < 0.05) respectively when compared to the GFP injected control of 1. This data confirmed EcR knockdown in An. funestus females injected with dsEcR. Data is representative of 2 biological replicates and normalised using an average of RPS7 and RPL19 reference genes. Expression levels calculated using relative quantification method (∆∆Ct). At each time point statistical significance was assessed with the unpaired student’s t-test. *p < 0.05, **p < 0.01. Error bars represent standard deviation. [file 12936_2022_4123_MOESM1_ESM.tif]

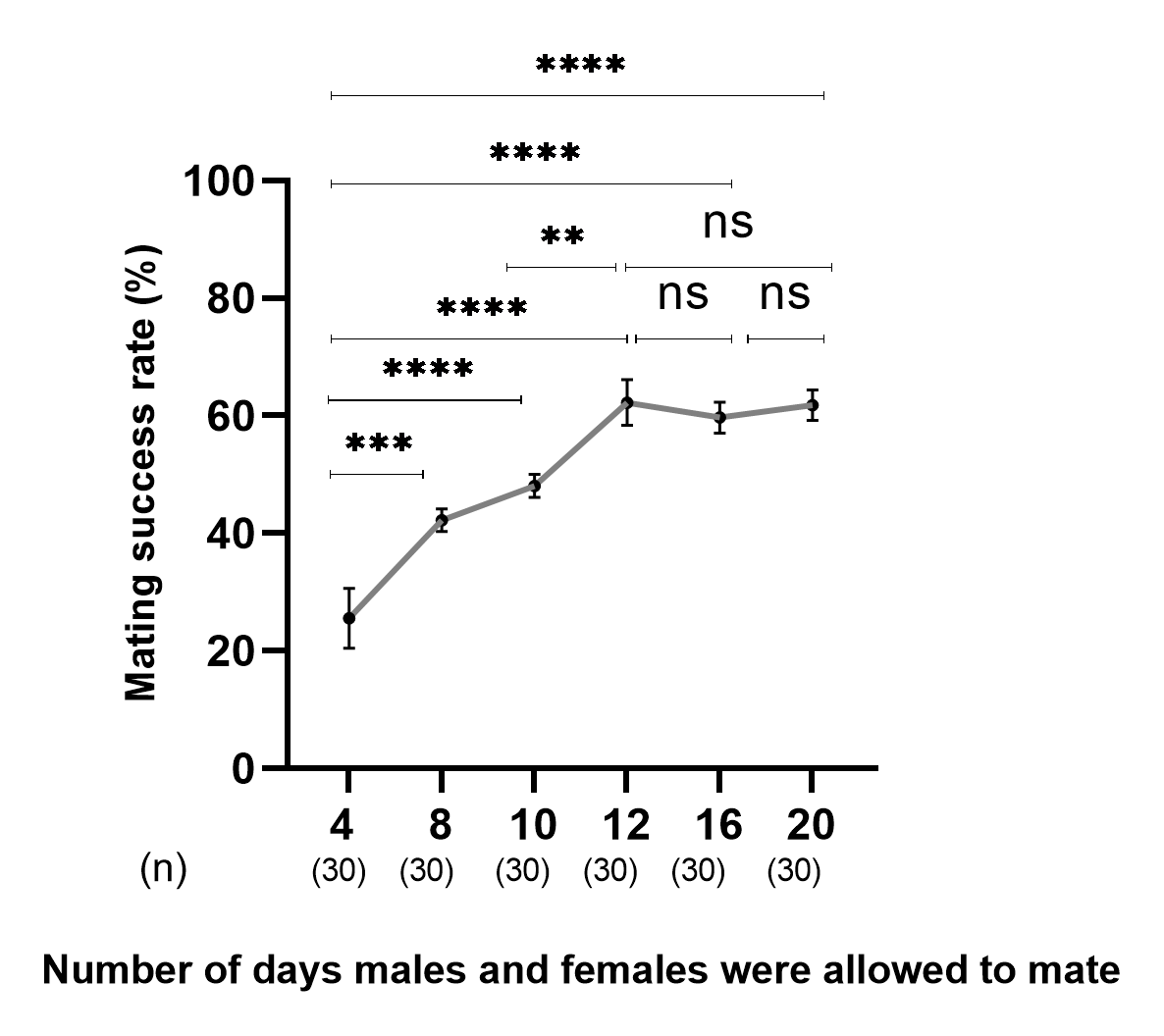

Supplement: Supplementary file 2 — Additional file 2: Figure S2. The highest mating success rate was achieved when An. funestus males and females are combined for 12 days after which no further increases are observed. The percentage mating success rate increased progressively until it reached its highest value of 62.2% after 12 days of mating. After this point, the mating success rate reached a plateau until day 20. Statistical significance was calculated using one-way ANOVA with Tukey’s post hoc analysis to correct for multiple comparison. Data represents the means of 3 biological replicates. Error bars represent standard deviation of means. ns = not statistically significant p > 0.05; ** = p < 0.01; *** = p < 0.001; **** = p < 0.0001. (n) = number of females per time point across 3 biological replicates. [file 12936_2022_4123_MOESM2_ESM.tif]

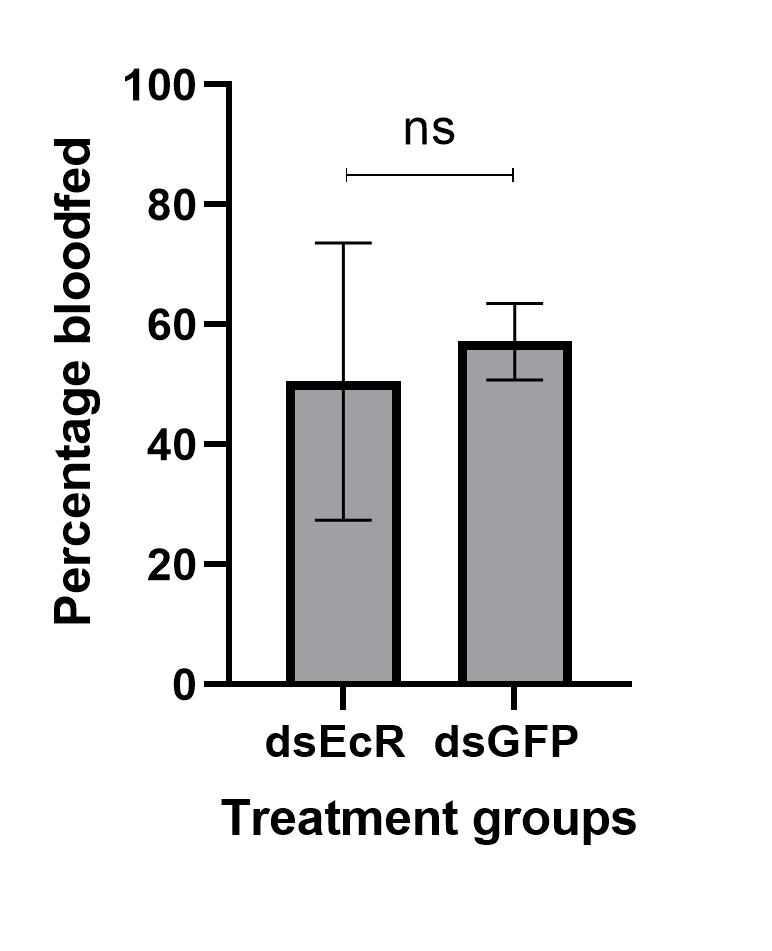

Supplement: Supplementary file 3 — Additional file 3: Figure S3. Blood feeding rates did not differ between treatment groups. Insignificant differences amongst treatment groups confirmed that the blood feeding rates did not influence any changes observed in the phenotypes of dsEcR treated An. funestus females (p > 0.05). Statistical significance calculated using an unpaired student’s t-test. Error bars represent standard deviation. ns = not statistically significant p > 0.05. [file 12936_2022_4123_MOESM3_ESM.tif]
