# Supplementary material for: The ecdysone receptor regulates several key physiological factors in Anopheles funestus
Source: Malar J. 2022 Mar 19;21:97. doi: 10.1186/s12936-022-04123-8 (PMC8934008; doi:10.1186/s12936-022-04123-8)
Supplement: Supplementary file 4 — Additional file 4: Table S1. Statistical significance between dsGFP and uninjected controls from the various biological assays conducted. [file 12936_2022_4123_MOESM4_ESM.docx]

**Supplementary table:**

**Table S1:** **Statistical significance between dsGFP and uninjected controls from the various biological assays conducted.**

| Figure | Data | Value of dsGFP control | Value of uninjected control | Statistical difference between dsGFP and uninjected controls | Statistically significant |
| --- | --- | --- | --- | --- | --- |
| 2 | Longevity | Median survival of 14 days (95% CI of ratio: 2.96 - 5.41) | Median survival of 21 days (95% CI of ratio: 0.12 - 0.23) | (*χ*^2^_(1,_ *_N_*_= 240)_ = 2.011, *p*=0.1561) | No |
| 3A | Number of eggs oviposited per mated female | 48.11 ± 4.12 | 42.54 ± 3.96 | (*t*(91) =0.1522 , *p* =0.8793) | No |
| 3C | Eggs developed/ not developed per mated female | 89% | 89% | p>0.9999 | No |
| 3D | Percentage fertility per female | 86% (76-93%) | 84%(75-90%) | (*t*(46) =0.5975 , *p* =0.5531) | No |
| 4 | *P. falciparum* infection intensity | 9.37 ± 1.17 | 6.97% ± 0.95 | (Mann-Whitney U= 2300, *p* = 0.5337) | No |
| 4 | *P. falciparum* infection prevalence | 69.49% (± 6.05) | 83.64% ± 5.03 | (*χ*^2^_(1,_ *_N_*_= 720)_ =0.2584, *p*= 0.6112) | No |
